# Case Report: Diagnostic Challenges in the Detection of a Mixed *Plasmodium vivax*/*ovale* Infection in a Non-Endemic Setting

**DOI:** 10.4269/ajtmh.20-0079

**Published:** 2020-04-20

**Authors:** Ha Thu Trang Nguyen, Fabrizio Romano, Rahel Wampfler, Konrad Mühlethaler, Egbert Tannich, Alexander Oberli

**Affiliations:** 1 Institute for Infectious Diseases, University of Bern, Bern, Switzerland;; 2 Pediatric Emergency Department, Inselspital, Bern University Hospital, University of Bern, Bern, Switzerland;; 3 Swiss Tropical and Public Health Institute, Basel, Switzerland;; 4 University of Basel, Basel, Switzerland;; 5 Bernhard Nocht Institute for Tropical Medicine, Hamburg, Germany

## Abstract

In clinical practice, mixed-species malaria infections are often not detected by light microscopy (LM) or rapid diagnostic test, as a low number of parasites of one species may occur. Here, we report the case of an 8-year-old girl migrating with her family from Afghanistan with a two-species mixed infection with *Plasmodium vivax* and *Plasmodium ovale*. This case demonstrates the significance of molecular testing in the detection of mixed-species malaria infections and highlights the importance of a detailed data analysis during the medical validation procedure to prevent underestimation of mixed-species infections. To our knowledge, this is the first case report of a two-species mixed infection comprising both *P. vivax* and *P. ovale* confirmed by LM and different real-time polymerase chain reaction (PCR) approaches.

## CASE

**Figure 1.**
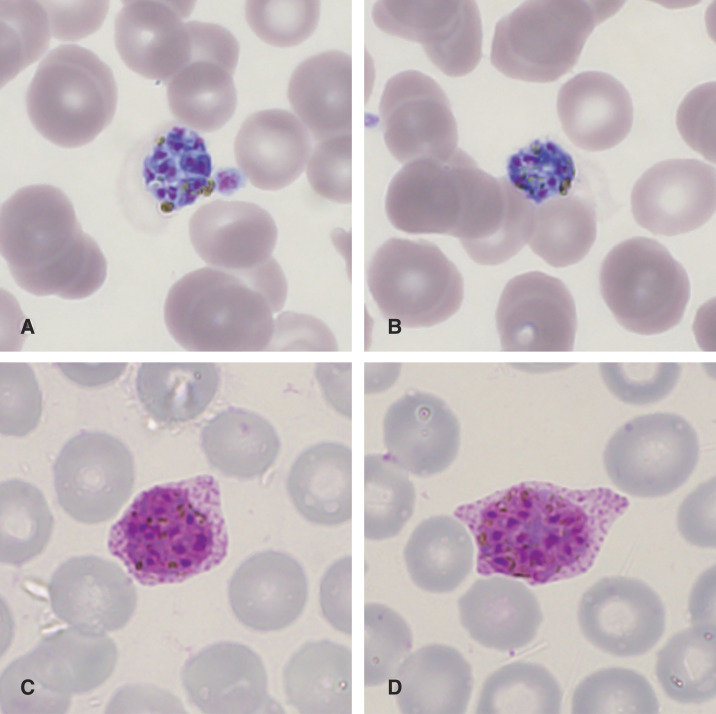
Light microscopy of a Giemsa-stained peripheral blood smear (10 × 100 magnification) of sibling 2. Besides characteristic *Plasmodium vivax* parasites (**C** + **D**), two mature schizonts with 13 (**A**) and nine (**B**) merozoites were observed. Note the fimbriated and oval-shaped infected erythrocytes with a cluster of dark brown pigment (**A** + **B**). This figure appears in color at www.ajtmh.org.

Six months after immigrating to Switzerland, four members of a seven-member Afghan family (aged 2, 8, 10, and 15 years) were admitted to the pediatric emergency ward with a history of intermittent fever and malaise. Since their arrival in Switzerland, all four children have had repeated fever attacks, initially every 1–2 weeks. Further consultations followed because of increases in fever frequency, at the time of presentation every 2–3 days, accompanied by headache, nausea, and musculoskeletal and abdominal pain. In the past, all siblings were already treated for malaria in Dschalalabad, Afghanistan. Detailed information about diagnosis, prescribed antimalarial drugs, point of time, and duration of therapy could not be determined. On examination, all children presented in maintained general condition. No fever focus could be identified, and no organomegaly was palpable. The demographic and clinical laboratory data of each of the four children are summarized in Table 1. The reoccurring fever symptoms and the partially reduced thrombocyte counts were compatible with a relapsing or recrudescent form of malaria. Examination of peripheral blood smears by light microscopy (LM) according to standard protocols showed *Plasmodium vivax*–infected erythrocytes for all four siblings, with 1.05–3.25% parasitemia, and rapid malaria antigen tests (BinaxNOW Malaria Test, Alere, Scarborough, ME) were all negative for *Plasmodium falciparum* and positive for pan-malaria antigen. For sibling 2, the LM revealed a *P. vivax* infection, but some schizonts showed features not typical for *P. vivax*. Very few schizonts comprised only eight merozoites, and some of the infected erythrocytes were oval, fimbriated, and slightly enlarged, thus resembling *Plasmodium ovale* schizonts (Figure 1A and B). However, the vast majority of schizonts were identified as *P. vivax* (Figure 1C and D). Retrospective analysis of ethylenediaminetetraacetic acid (EDTA) blood of sibling 2 using a *Plasmodium* species–specific multiplex real-time PCR (Fast Track Diagnostics (FTD) Malaria Differentiation, Fast Track Diagnostics, Sliema, Malta) revealed a *P. vivax* infection with a cycle threshold (Ct) value of 11.6. Subsequent in-detail analysis of the real-time PCR quantification data further showed an exponential/sigmoidal amplification curve for *P. ovale*, but the fluorescent signal was weak and below the minimum relative fluorescent units (RFU) peak height. To further investigate whether sibling 2 has *P. vivax*/*P. ovale* mixed infection or a single species infection, multiplex real-time PCR and monoplex real-time PCR with species-specific oligonucleotides were performed in the malaria reference laboratories of Switzerland (Swiss TPH) and Germany (Bernhard Nocht Institute for Tropical Medicine, Hamburg, Germany). Amplification of both *P. vivax* and *P. ovale* DNA by monoplex real-time PCR clearly indicated a mixed infection with *P. vivax* (Ct values 18.6 and 12.0) as the main species and *P. ovale* (Ct values 33.6 and 29.0) as the minor species. Subsequent characterization of the *P. ovale* subspecies by *P. ovale*–specific PCRs, based on a conserved region between *P. ovale curtisi* and *P. ovale wallikeri*, followed by sequencing of the amplified DNA, revealed sibling 2 being positive for *P. ovale curtisi*.^1^ All patients were initially treated with artemether/lumefantrine for 3 days (weight-based dose according to the international guidelines) followed by primaquine 0.5 mg/kg/dose and chloroquin 2.5 mg/kg/dose both once daily for 14 days to eradicate possible hypnozoites and to prevent relapses. There was a complete recovery from clinical symptoms for all four siblings and a normalization of blood thrombocyte counts.

**Table 1:** Patient demographic and clinical laboratory data

	Sibling 1	Sibling 2	Sibling 3	Sibling 4
Age (years)	2	8	10	15
Time of diagnosis (since arrival) (months)	6	9	7	6
Parasitemia (%)	3.25	1.5	1.4	1.05
BinaxNow rapid diagnostic test	non-*P. f.*	non-*P. f.*	non-*P. f.*	non-*P. f.*
Parasite identification (microscopic)	*P. vivax*	*P. vivax* (*Plasmodium ovale*)	*P. vivax*	*P. vivax*
FTD real-time PCR cycle threshold-value (*P. vivax*)	19.0	11.6	16.4	15.4
Hemoglobin (10–16 g/dL)	8	11.9	1.9	10
Thrombocytes (150–450 × 103/mm^3^)	121	107	131	136
Leukocytes (3.0–12.5 × 103/mm^3^)	9.7	6.1	6.36	4.72
C-reactive protein (< 3 mg/L)	46	32	7	50
Alanine transaminase (U/L)	21	19	24	14

*P. f.* = *Plasmodium falciparum*; *P. vivax* = *Plasmodium vivax*.

## DISCUSSION

Several species of the genus *Plasmodium* are known to cause malaria in humans, and clinical courses and severity of the disease differ according to the species. Therefore, an accurate and fast laboratory diagnosis is critical to administration, management, and treatment of malaria patients. *Plasmodium vivax* is the major cause of malaria morbidity in Afghanistan, and primaquine is in the treatment policy of Afghanistan although there is limited access to effective radical cure therapy and G6PD testing.^2^ As in Afghanistan *P. vivax* has a long latency period before relapse, the diagnosis of malaria should be considered in all febrile migrants from this region.^2^ In non-endemic regions, first-line malaria diagnosis commonly consists of a combination of LM (thick and thin blood smears) and rapid diagnostic test (RDT).^3^ Although LM of Giemsa-stained blood films is reasonable, fast, and inexpensive, differentiation of *Plasmodium* species and quantification of parasitemia require well-trained personnel. The limit of parasite detection (LoD) of LM using thick blood films has been estimated to be about 50 parasites per microliter, whereas RDTs revealed good sensitivity of at least 200 parasites per microliter for the detection of *P. falciparum*, but the sensitivity for other *Plasmodium* species is limited.^4^ However, for the detection of mixed infections with two or more *Plasmodium* species, the combination of malaria microscopy and RDTs for the initial diagnosis has a relatively low sensitivity.^5^ Therefore, mixed infections have a high risk of not being detected. This has several reasons: 1) the morphological species differentiation of early-stage parasites (ring forms) can be misleading; 2) the parasitemia of the less abundant species may be below the LoD of LM; 3) after the initial diagnosis of the abundant species, the low abundant species might be overseen; and 4) one species is absent in the blood sample persisting as hypnozoites in the liver (in the case of *P. vivax* and *P. ovale*). Recent reports using molecular methods in parasite detection indicated reduction in costs and therapeutic turnaround time as well as higher prevalences of mixed-species infections compared with detection by LM.^6,7^ In the case of sibling 2, real-time PCR analysis confirmed a *P. vivax* infection with a high parasite load, as reflected by the relatively low Ct value. In-depth analysis of the melting curves revealed a fluorescent signal for *P. ovale* below the minimum RFU peak height. There is no standard rule for establishing minimum RFU thresholds for data interpretation, and this is one important aspect of the medical validation procedure of the data generated by the diagnostic laboratory. As there is no clear consensus on standardized PCR-based methods, diagnostic laboratories are strongly encouraged to follow the WHO external quality assurance scheme for malaria nucleic acid amplification testing.^8^ When the amount of DNA is very low, it is difficult to differentiate a true level RFU peak from signal noise or artifacts of any origin. Especially, in a multiplex real-time PCR setting with a mixed-species infection, RFU of the low abundant species might be suppressed.^9^ Subsequent monoplex real-time PCR with two different sets of *P. ovale* species–specific oligonucleotides confirmed a *P. vivax*/*P. ovale* mixed infection with a *P. ovale curtisi*^1^ subtype*.* To the best of our knowledge, this is the first documentation of a two-species mixed infection comprising both *P. vivax* and *P. ovale* detected by LM and confirmed by real-time PCR. So far, mixed plasmodia infections comprising either *P. vivax* or *P. ovale* have only been described in combination with either *P. falciparum* and/or *Plasmodium malariae* as double- or triple-species infections. Moreover, the *P. vivax/P. ovale* species combination was only found in triple (*P. falciparum*, *P. vivax*, and *P. ovale*) or quadruple infections (*P. falciparum*, *P. vivax*, *P. ovale*, and *P. malariae*).^10,11^ This might have different reasons. *Plasmodium ovale* may easily be confounded by other species, as usually parasitemia is lower than in *P. falciparum* or *P. vivax* infection. Moreover, species identification by LM requires experienced microscopists as the morphology of the two species resembles each other and, thus, may be difficult to differentiate in particular in cases of low parasitemia. Furthermore, it is discussed that simultaneous infection with different *Plasmodium* species can result in suppression of PCR for one of the species.^9^ Thus, it might be possible that *P. ovale* prevalence and parasitemia are consistently low for appropriate evaluation. Another explanation of the rarity of *P. vivax*/*P. ovale* mixed infection may be that these two species compete for invasion of host cells as both species prefer to invade reticulocytes. Because of the increased availability and increased use of species-specific malaria real-time PCR, it becomes evident that results obtained by LM are at least in part inaccurate, especially in cases of non-*falciparum* and mixed infections. A recent study combining LM and real-time PCR revealed that molecular methods consistently detect higher prevalences for all malaria species infecting humans, with the highest increase in *P. malariae* and *P. ovale*.^12^ In all settings, in which molecular diagnostic tools such as real-time PCR are established and maintained, it is likely that real-time PCR will extend the diagnostic possibilities in case of incongruent LM and RDT not only to examine the presence of low-level parasitemia, but also to detect suspected mixed-species infections, as it is a rapid, semiquantitative, sensitive, and specific diagnostic method to detect and differentiate malaria parasites. This is in accordance with a recent report, in which the good diagnostic performance of real-time PCR in non-endemic settings was confirmed and proposed to be included in the panel of diagnostic tools for imported malaria.^13^ This case highlights the importance of molecular testing and detailed data analysis in the medical validation process for the detection of a mixed-species malaria infection in a non-endemic region.
